# Editorial: Reviews in insect immune responses: 2022

**DOI:** 10.3389/fimmu.2024.1414382

**Published:** 2024-06-21

**Authors:** Qiuning Liu

**Affiliations:** ^1^ Jiangsu Key Laboratory for Bioresources of Saline Soils, Jiangsu Synthetic Innovation Center for Coastal Bio-agriculture, Yancheng Teachers University, Yancheng, China; ^2^ Jiangsu Provincial Key Laboratory of Coastal Wetland Bioresources and Environmental Protection, School of Wetlands, Yancheng Teachers University, Yancheng, China

**Keywords:** insect immunity, pattern recognition receptors, immune-related genes, antimicrobial peptides, miRNAs, immune signaling pathways

Insects, with their vast diversity and ecological significance, have long been a focal point of biological research (1). The intricate and multi-layered defense mechanisms of insects against a myriad of pathogens have been a focal point of scientific inquiry for decades (2). Among the myriad of topics explored within entomology, the study of insect immune responses stands out as a critical area of investigation (3). This editorial introduces the Research Reviews in Insect Immune Responses for 2022, a Research Topic that delve into the complexities of how insects defend themselves against a multitude of pathogens and the implications of these defense mechanisms for both basic and applied sciences. The aims of this Research Topic are to encapsulate the diversity of research within the field of insect immunity and to provide a platform for the dissemination of recent findings that have the potential to transform our approach to insect health and disease management. The articles within this Research Topic span a wide array of topics, from molecular and cellular level analyses to whole-organism and population-level studies, reflecting the multidisciplinary nature of insect immunology.

Insects and bacteria engage in a dynamic crosstalk that influences various aspects of life due to their widespread distribution and the role of insects as disease vectors (4). miRNAs are known for their dynamic expression and diverse targets, which allow them to control key physiological processes, including the innate immune response of insects (5). Mahanta et al. presented the relationship between miRNA dysregulation during bacterial infections and the subsequent progression of the disease. Recent findings highlight the importance of miRNAs in bacterial infections, where they modulate the host’s immune system and contribute to resistance mechanisms. This review discusses the relationship between miRNA dysregulation during bacterial infections and the subsequent progression of the disease.

Abbas et al. synthesized recent findings on the role of miRNAs in insect immunity, particularly in the context of bacterial infections. It underscores the importance of miRNAs in shaping the host’s immune response and the potential of these molecules as targets for developing novel strategies to enhance insect resistance to pathogens. It particularly underscores the impact of miRNAs on major signaling pathways such as Toll, IMD, and JNK, which are central to the insect immune response. They also examined the biological functions of miRNAs in immune regulation and points out the existing gaps in our understanding of their role in insect immunity.

Pattern Recognition Receptors (PRRs) are integral to the immune system’s ability to detect invading pathogens by recognizing Pathogen-Associated Molecular Patterns (PAMPs). PRRs such as Peptidoglycan Recognition Protein (PGRP), Gram-Negative Binding Protein (GNBP), β-1,3-Glucan Recognition Protein (βGRP), C-Type Lectin (CTL), and Scavenger Receptor (SR) are crucial for initiating defense responses (6). Recent research has also highlighted the role of Damage-Associated Molecular Patterns (DAMPs), which, when extracellularly exposed, can signal for immune responses beyond their normal cellular functions (7). Zhao et al. investigated the various PRRs in Lepidoptera and their roles in pathogen recognition, as well as the interaction between PRRs and DAMPs in the immune response. It also explores the relationship between PRRs and the immune evasion strategies of pathogens. The findings indicate that PRRs may have a more extensive role in insect innate immunity than previously understood, potentially recognizing a wider array of signaling molecules. This knowledge enhances our understanding of the Lepidoptera immune system and could inform strategies for disease management and pest control in these species.

The insect gut plays a pivotal role in defending against a variety of pathogens and harmful substances, making it a key site for immune responses (8). It is composed of unique compartments, including the peritrophic membrane, mucus layer, and microvilli, which contribute to physiological processes and immunity (9). Khan et al. demonstrated that the gut microbiota also plays a significant role, influencing signaling pathways and contributing to gut homeostasis by producing essential vitamins and minerals. When insects encounter pathogens or harmful substances, specific immune signaling pathways within the gut are activated. These pathways, such as the IMD, Toll, JAK/STAT, Duox-ROS, and JNK/FOXO, are responsible for producing antimicrobial peptides (AMPs) that help maintain gut balance. Khan et al. provided valuable insights into the structure and function of the insect gut, the role of commensal microorganisms, and the signaling pathways involved in immune responses and recovery. Understanding these mechanisms is essential for developing strategies to enhance insect health and resistance to diseases.

Insects, making up a significant portion of the world’s fauna, are often targeted by entomopathogenic fungi (EPF), which serve as effective biopesticides (10). The interaction between insects and EPF is a complex life-and-death struggle that unfolds across various stages of infection (11). Ma et al. summarized the sophisticated defense mechanisms insects have evolved to counter EPF infections and the strategies EPF employ to overcome these defenses. Ma et al. provided new insights for developing more effective fungal insecticides and pest management practices. Understanding these interactions is crucial for harnessing EPF as a biological control agent, potentially reducing reliance on chemical pesticides and promoting more sustainable pest control methods.

In conclusion, this editorial introduces a Research Topic that encapsulate the current state of research in insect immunology, providing a comprehensive overview of the field and setting the stage for future advancements. The Research Reviews in Insect Immune Responses: 2022 serve as a preface to a rich and diverse body of work that is shaping our understanding of these complex and fascinating defense systems. This Research Topic offers a rich tapestry of insights into the world of insect immune responses, reflecting the depth and breadth of current research. We hope that these reviews will inspire continued curiosity, collaboration, and innovation in the pursuit of knowledge in insect immunology.

## Author contributions

QL: Writing – original draft, Writing – review & editing.
